# Defining the variety of cell types in developing and adult human kidneys by single-cell RNA sequencing

**DOI:** 10.1038/s41536-021-00156-w

**Published:** 2021-08-11

**Authors:** A. Schumacher, M. B. Rookmaaker, J. A. Joles, R. Kramann, T. Q. Nguyen, M. van Griensven, V. L. S. LaPointe

**Affiliations:** 1 MERLN Institute for Technology-Inspired Regenerative Medicine, Department of Cell Biology–Inspired Tissue Engineering, Maastricht University, Maastricht, The Netherlands; 2 Department of Nephrology, University Medical Center, Utrecht, The Netherlands; 3 Institute of Experimental Medicine and Systems Biology, RWTH Aachen, Aachen, Germany; 4 Department of Internal Medicine, Nephrology and Transplantation, Erasmus Medical Center, Rotterdam, The Netherlands; 5 Department of Pathology, University Medical Center, Utrecht, The Netherlands

**Keywords:** Cell biology, Kidney, Regenerative medicine, Tissue engineering

## Abstract

The kidney is among the most complex organs in terms of the variety of cell types. The cellular complexity of human kidneys is not fully unraveled and this challenge is further complicated by the existence of multiple progenitor pools and differentiation pathways. Researchers disagree on the variety of renal cell types due to a lack of research providing a comprehensive picture and the challenge to translate findings between species. To find an answer to the number of human renal cell types, we discuss research that used single-cell RNA sequencing on developing and adult human kidney tissue and compares these findings to the literature of the pre-single-cell RNA sequencing era. We find that these publications show major steps towards the discovery of novel cell types and intermediate cell stages as well as complex molecular signatures and lineage pathways throughout development. The variety of cell types remains variable in the single-cell literature, which is due to the limitations of the technique. Nevertheless, our analysis approaches an accumulated number of 41 identified cell populations of renal lineage and 32 of non-renal lineage in the adult kidney, and there is certainly much more to discover. There is still a need for a consensus on a variety of definitions and standards in single-cell RNA sequencing research, such as the definition of what is a cell type. Nevertheless, this early-stage research already proves to be of significant impact for both clinical and regenerative medicine, and shows potential to enhance the generation of sophisticated in vitro kidney tissue.

## The need for well-characterized renal cell types

A detailed understanding of the variety of cell types within the healthy kidney and their molecular composition will benefit scientists aiming to treat patients with kidney failure. The ideal treatment is kidney transplantation from a healthy immune-matching donor. However, donor kidney availability is far from meeting its demand, and waiting times are long so that many patients die while waiting for transplantation. There is great demand for new therapies, as current treatment methods, such as dialysis, do not provide all essential functionalities of a kidney and are not long-term solutions^1^. For example, toxins are insufficiently removed and the sodium and fluid homeostasis are distorted by intermittent treatment, while the metabolic and endocrine functions are completely neglected^2,3^. Dialysis can replace many functions of the kidney but is associated with high morbidity, mortality, reduced quality of life, and high costs. Currently, the prevalence of patients with chronic kidney disease is as high as 9.1% with an age-dependent mortality rate of 1.5–6.3%^4^. Recent projections indicate that by 2030, nearly 5.5 million people worldwide could depend on renal replacement therapy^5^

Due to the aforementioned shortcomings, clinicians and scientists aim to understand hurdles such as kidney development and regeneration, factors involved in kidney failure and disease, how to improve survival on dialysis and prevent donor tissue rejection. Furthermore, joint forces of (tissue) engineers, material scientists, and (developmental) biologists are working worldwide on artificial and bioengineered kidneys as a replacement for current dialyses. They have made major advances in the de- and re-cellularization of rodent and human kidneys^3^, renal tubule assist devices^6^, lightweight miniaturized kidneys^7^, and implantable bioartificial devices^7^ and tissues^8^, among others. All these approaches would benefit from a greater understanding of the cellular and molecular composition of developing an adult kidney.

Creating kidneys in vitro is, however, a challenging task that involves the coordination of complex cellular and molecular events^9^. Starting in 2014, major breakthroughs were published on the creation of miniaturized self-assembled kidney tissue (organoids) in vitro from induced pluripotent stem cells^10–12^. These organoids contain various renal cell types, patterned similarly to in vivo tissue. Nevertheless, they are incomplete and essential questions remain, such as: if the developmental lineage steps are being followed, why do some cell types emerge (e.g., podocytes and endothelial cells) while others do not (e.g., mesangial cells (MCs) and parietal epithelial cells (PECs), why does the tissue deteriorate after a few weeks in culture, and why does it not mature beyond the first trimester^13,14^, unless transplanted in vivo^15^. Finally, how can we achieve and maintain a complex 3D architecture of a nephron, tubulo-interstitium and vasculature? In order to answer such questions and to develop more mature tissues for transplantation, there is a greater understanding needed of the large variety of renal cell types, their plasticity, lineage, cell state, phenotype switching, and functionality.

## Divergent numbers of renal cell types

Outstanding research has been conducted in the past decades to unravel mammalian kidney development and nephrogenesis and to characterize adult kidneys^16–21^. However, the extraordinary developmental complexity has made scientists across fields struggle to determine the number of renal cell types and to replicate these cell types in vitro. Heterogeneity is a regular term appearing in these publications, being a clear indication of one of the major difficulties in kidney research. So far, the field generally agrees on certain heterogeneity between individuals such as the timing of kidney development (e.g., cessation of nephrogenesis from 32^22^ to 37 weeks^23^ of gestation), numbers of nephrons (210,000–1,825,000 nephrons per kidney with an average of 600,000–800,000^24,25^), cell numbers per segment (e.g., 431–746 podocytes per adult glomerulus^26^) and anatomical differences (e.g., number of renal papillae^27^). However, there is still little consensus on the number or range of distinct renal cell types; neither in humans nor in rodents.

More than 18−26 cell types have been described in mammalian mature kidneys^21,28–36^, of which at least half are epithelial and/or located within nephrons^37^. While the source of these numbers is challenging to trace, possible reasons for the range can include the unclear definition of what is a distinct cell type, incomplete knowledge of cell-specific markers, technical limitations, a certain degree of variability in healthy subjects, and the difficulty of defining cell identities and distinguishing cell types. Additionally, inter-species differences could underlie these ranges, as often no species is mentioned apart from “mammalian kidneys”. Clearly, part of the challenge is a lack of consensus on the definition of what constitutes a distinct cell type and the resolution of cell-specific characteristics before we can elucidate the renal cellular complexity in humans in a comprehensive way.

## Defining cell identity, plasticity, and maturity

It remains questionable if it is possible to state a specific number of renal cell types and which techniques would lend the most appropriate data to do so. A specific cell number would imply a clear-cut definition of what is considered a distinct cell type. While researchers try to define cellular plasticity^38^, this central, measurable definition of “cell type” has not yet been determined. Earlier, an evolutionary perspective was suggested, which states that a cell type can be defined by a core regulatory complex (CoRC), which is a set of transcription factors and their interacting factors^39^. In a recent publication, Morris^40^ proposes a more complex viewpoint made of three major components: (1) phenotype and function (physical, molecular and functional features), (2) lineage, and (3) state (changes in cell state in response to stimuli). The general idea is that cell types cannot be clearly identified by solely assessing only one of the three components.

Many existing techniques for cell type detection and characterization have technical limitations. Initially, histological stains were used, followed by techniques like immunostaining, (fluorescent) in situ hybridization (FISH), and flow cytometry. However, these techniques are biased by the existing knowledge of cellular markers, are by definition not designed to discover larger scales of novel markers, and are limited in the number of markers that can be simultaneously analyzed. This makes it technically impossible to assess a single cell on all three components of cellular identity. More recently, bulk RNA sequencing allowed the exploration of novel markers by providing average gene expression across a large population of cells^41–43^. However, the expression of low abundance genes is underrepresented in bulk sequencing and cellular lineage cannot be determined^41,44^. Now, in the era of single-cell RNA (scRNA) sequencing, lineages can be determined and rare gene detection is possible. The heterogeneity within cell populations can be detected in high throughput^41,42^, as can the profiling of cell states and transitions during differentiation^41,45–47^. Therefore, while not free of limitations, scRNA sequencing is a promising technique to answer the long-standing question of the number of renal cell types.

This review aims to investigate the variety of renal cell types in the developing and adult human kidney by discussing common and conflicting findings of scRNA sequencing studies. Given our focus on regenerative medicine and tissue engineering, we excluded publications on renal pathology and drug testing. Briefly, the review first addresses the major stages of nephrogenesis to discuss the cellular lineages within these stages, which are important for researchers aiming to replicate development in vitro. Subsequently, findings on the cellular variety of adult kidneys will be discussed. The discovery of novel cell types and markers, subpopulations, intermediate cell states, segment transitions, as well as phenotype-specific expression patterns, and developmental trajectories will be covered. Finally, shortcomings and opportunities of scRNA sequencing will be discussed in the context of kidney research.

## Main analysis

### Structural development of the human metanephric kidney

**Fig. 1:**
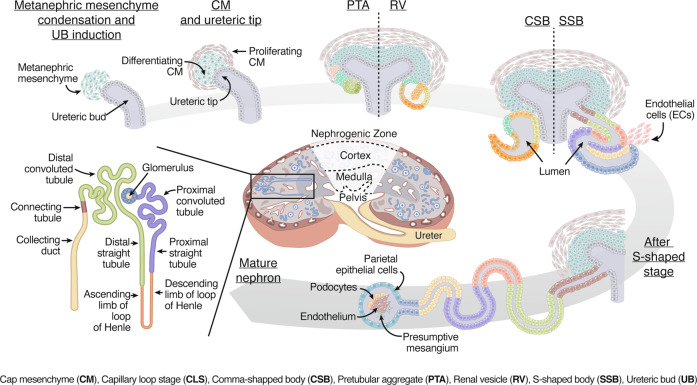
Schematic representation of nephrogenesis, starting with the interaction of the cap mesenchyme (CM) and ureteric bud (UB) in the nephrogenic zone. Differentiation takes place along morphologically determined stages known as pretubular aggregate (PTA), comma-shaped body (CSB), renal vesicle (RV), S-shaped body (SSB), and capillary loop stage (CLS) nephron. The mature nephron is located throughout the cortex, whereas the Loop of Henle and collecting duct extend into the medulla.

Here we highlight the major stages of development, knowledge needed for the remainder of this review. Development of the human metanephric kidney begins around four weeks of gestation^17^ from a close interaction of the metanephric mesenchyme (MM) and the ureteric bud (UB) (Fig. 1). While both MM and UB derive from the intermediate mesoderm, it is in the MM where nephron progenitor cells (NPCs) differentiate into nephron tubules, the glomerulus, and the renal stroma; whereas the UB gives rise to the collecting duct and ureter^18^. Simultaneous to the mesenchyme differentiation, UB differentiation and proliferation occur on the tip of the UB and lead to branching (bifurcation) into a tree-like pattern with later extensive elongation of the ureteric trunk^48,49^. The MM undergoes morphological changes during differentiation characterized as pretubular aggregate (PTA), renal vesicle (RV), comma-shaped bodies (CSB), and subsequently S-shaped bodies (SSB) before entering the capillary loop stage (CLS)^16,50,51^. The first SSB is detected around Carnegie stage (CS) 18–19^17^, from which further differentiation occurs.

Cells of the distal nephron segment start invading the collecting duct epithelium at the SSB stage to connect the nephron to the collecting duct^52^. Subsequently, endothelial cells start to invade the proximal segment of the SSB, initiating the CLS. This stage is further characterized by the development of the vascular system, including the glomerular capillaries, arteries, veins, and the appearance of the primitive loop of Henle (LoH)^53^. The first generation of glomeruli appears to be mature around week 9^54^ and shortly after, glomerular filtration begins^55^. Eventually, 8–12 generations of glomeruli are formed, leading to nephrons in different developmental stages. Nephrogenesis ceases before birth, by week 38–41^23^.

Nephrogenesis is further supported by the surrounding interstitium and extracellular matrix^56^. The term interstitium describes a tissue with a variety of cell types such as renal fibroblasts, pericytes, and cells with endocrine functionalities (renin- and erythropoietin-producing cells), with possible distinct origins^57^. Although the discussion of the origin of renal interstitium is ongoing, one perspective is that it emerges early in nephrogenesis from a FOXD1^+^ subpopulation of the MM^57,58^.

### Renal cell type discoveries by single-cell RNA sequencing

**Fig. 2:**
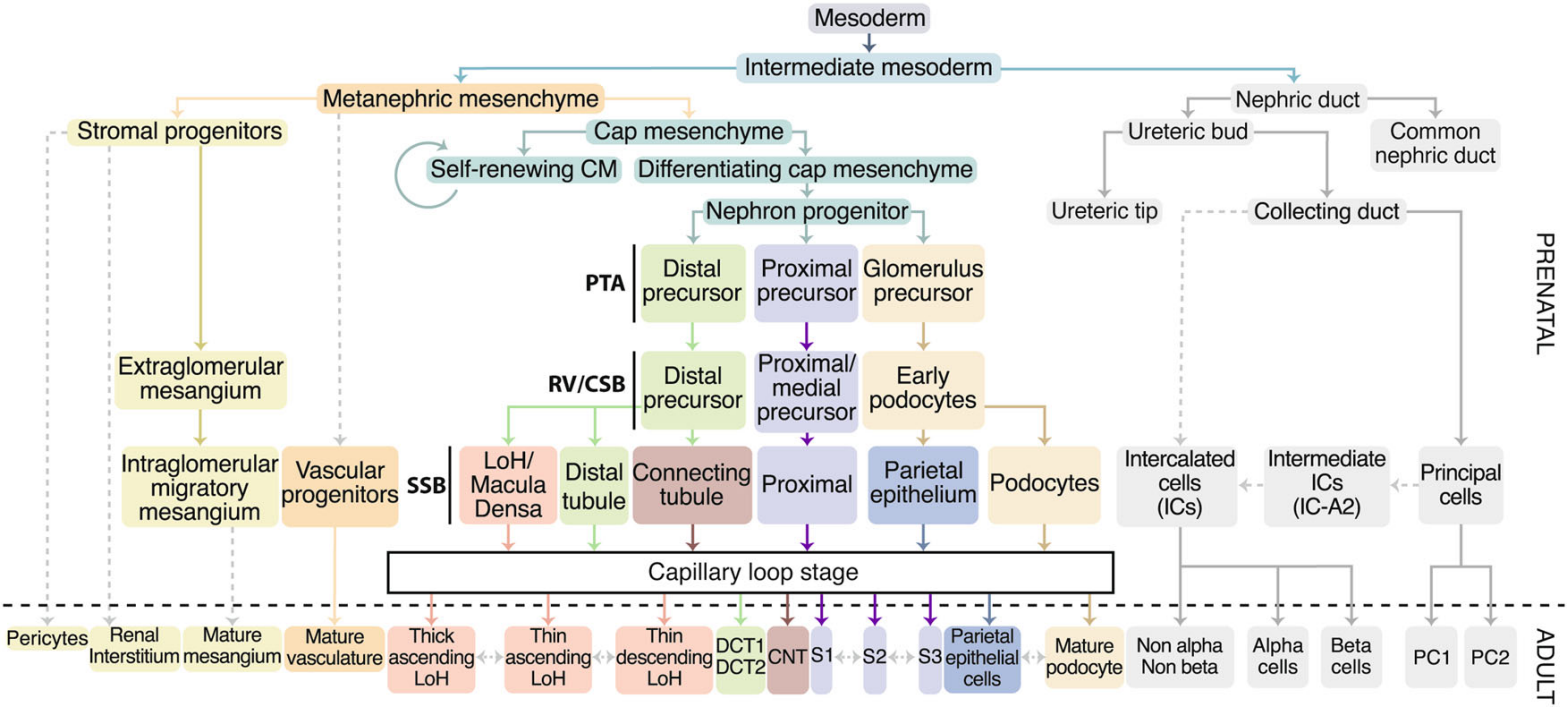
Lineage tree of renal cell types in development based on non-scRNA sequencing and scRNA sequencing literature. Dotted arrows indicate possible lineage relationships, which are not fully proven or not highlighted in the discussed scRNA sequencing publications. Pretubular aggregate (PTA), renal vesicle (RV), comma-shaped body (CSB), S-shaped body (SSB), Loop of Henle (LoH), connecting tubule (CNT), distal convoluted tubule 1 and 2 (DCT1/2), segment 1/2/3 (S1/S2/S3), intercalated cells (ICs), principal cells (PCs).

**Fig. 3:**
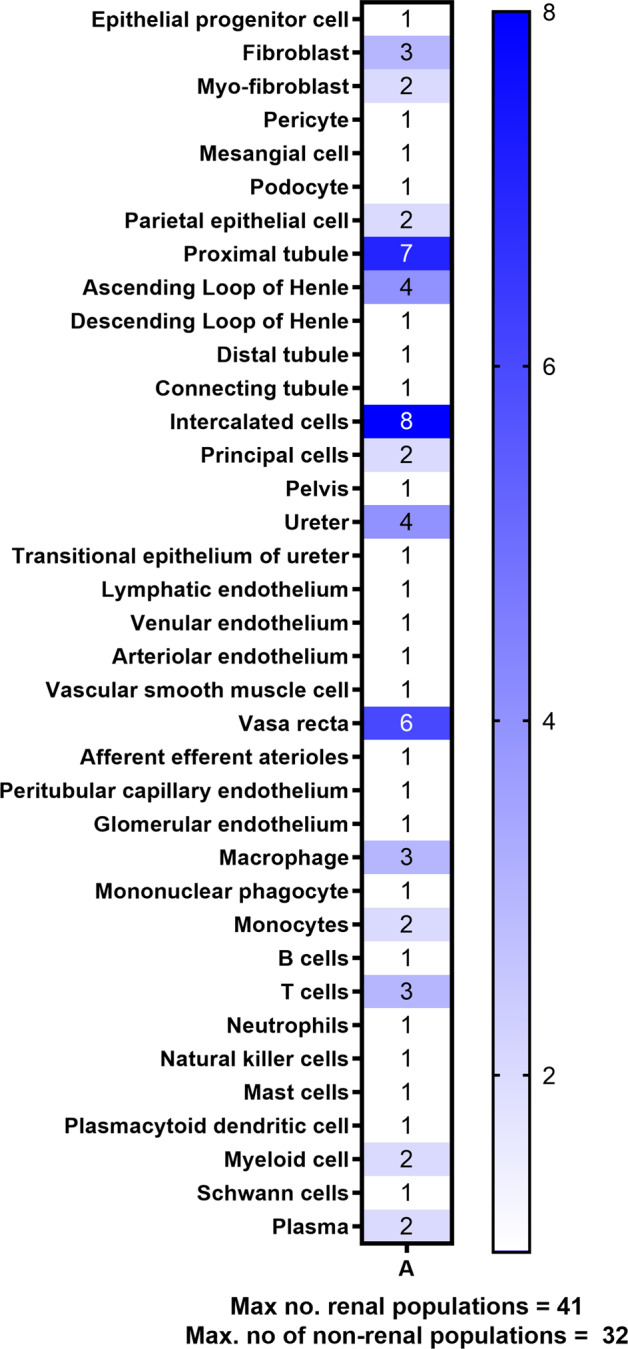
The variety of different renal cell populations identified by single-cell RNA sequencing to date. All cell types detected (accumulated) by all discussed scRNA sequencing publications. The number indicates the highest number of clusters distinguished by one or more of the discussed publications and with this, the total sum of populations was calculated. For the adult kidney, 41 renal and 32 non-renal populations were detected.

Hereinafter, we highlight discoveries from scRNA studies on fetal and adult human renal tissue and contrast the findings with earlier literature using techniques like histology, immunostaining, and FISH. We aim to elucidate the divergent numbers of clusters/cell types of the different scRNA and single nucleus RNA sequencing studies performed on human renal tissue to date, as summarized in Table 1 and Supplementary Table 2, and abbreviated as “scRNA sequencing papers” throughout this publication. Given the comprehensive topic, we decided to focus on kidney cells derived from the same intermediate mesoderm progenitor (excluding interstitial cells (ICs)) and summarize all findings in an updated lineage tree in Fig. 2. Consequently, the discussion of the variety of immune cells, vascular and blood cells present in developing and adult kidney goes beyond the scope of this review; however, all findings of these cell types are summarized in Fig. 3 and Supplementary Table 1.

**Table 1:** Donor information and detected cell clusters from single-cell RNA sequencing studies of healthy human kidney tissue.

Publication	Donor age (fetal in weeks; adult in years)^a^	Tissue source	Number of clusters	Number of clusters of metanephric mesenchyme lineage
Young, et al.^67^	Fetal (8, 9)	Whole kidneys	7	6
Menon, et al.^61^	Fetal (12.4, 15, 15.7, 16.4, 18.8)	Whole kidneys	11	18
Lindstrom, et al.^64^	Fetal (16)	Nephrogenic zone	12/15	10
Wang, et al.^68^	Embryonic/fetal (7–10, 13, 19, 22, 24, 25)	Whole kidneys	13	10
Combes, et al.^89^	Fetal (16)	Nephrogenic zone	16	13
Lindström, et al.^78^ (preprint)	Fetal (14–17)	Nephrogenic zone	18	18
Stewart, et al.^88^	Fetal (7 [F], 8 [M], 9 [F], 12 [M], 13 [F], 16 [F])	Whole kidneys	21	11
Tran, et al.^65^	Fetal (15, 17)	Inner and outer cortex sections	21	18
Hochane, et al.^59^	Fetal (9 [M], 11 [M], 13 [F], 15 [F], 16 [M], 18 [F])	Whole kidneys	22	19
Lindstrom, et al.^66^	Fetal (17)	Nephrogenic zone	22	15
Liao, et al.^100^	Adult (57–65 [2 × M + 1 × F])	Whole kidney sections	10	8
Wu, et al.^118^	Adult (70 [M])	Biopsy	16	6
Wu, et al.^104^	Adult (62 [M])	Cortex	17	7
Young, et al.^67^	Adult (72)	Interface or region-specific biopsies	19	10
Stewart, et al.^88^	Adult (44–72 [M + F])	Cortex, medulla, pelvis biopsy	25	12
Sivakamasundari, et al.^101^ (preprint)	Adult (62–66 [M + F])	Resection samples	27	11
Kuppe, et al.^86^	Adult (50–84)	Healthy tumor nephrectomy tissue	27	23
Lake, et al.^103^	Adult (>50 [M + F])	Cortex and medulla	30	19

^a^Biological sex (M = male/F = female) is indicated when known.

A more detailed table can be found in the supplementary files (Supplementary Table 2).

### Pseudo-time trajectory follows the developmental flow

Pseudo-time analysis is a computational method to establish a dynamic process experienced by cells and arranges these cells based on their progression through this process. Pseudo-time trajectory analysis suggested that the identified kidney cells generally seem to follow the known developmental stages from NPCs, CSB, and SSB to mature, differentiated cell types^59^. Interestingly, this analysis did not describe CLS as a separate stage during differentiation. The identified literature on fetal kidney (Table 1) agreed that podocytes emerge first from the SSB nephron progenitors, followed by proximal and distal tubules, and finally LoH. This confirms earlier knowledge on the timing of nephron patterning^60^, where proximal, distal segments, and podocytes followed distinct lineages, and the distal tubule further differentiated into LoH and the connecting tubule (CNT). To our knowledge, the early podocyte emergence has, however, not been described before the scRNA sequencing era. Additionally, Menon, et al.^61^ distinguished the UB, stroma, and nephron as three distinct developmental trajectories. Although podocytes in the same study had strong stress-related signaling, possibly a consequence of their dissociation, pseudo-time indicated an extraordinarily complex differentiation trajectory^59^. Needless to say, more studies are needed to confirm these findings to exclude interindividual differences and dissociation biases. Future research could consider reducing dissociation artifacts by, for instance, the addition of cold-active proteases^62^.

### The balance of self-renewal and differentiation in early kidney development

The cap mesenchyme (CM) is considered the renal stem cell niche from which, in interaction with the ureteric epithelium, the mammalian kidney develops. Throughout development, the CM is maintained to repeatedly supply NPCs to undergo mesenchymal-to-epithelial transition (MET) and form the epithelial RV^63^. The number of different cell types at this stage has not yet been elucidated and scRNA sequencing research reveals that this will be a challenging task.

CM and NPCs populations were detected to various extents in the scRNA sequencing publications, with most papers describing one or the other, but not both populations (Supplementary Table 1). This could be explained by the different fetal ages. Studies of week 15–17 fetuses^64–66^ might be less likely to detect CM clusters, since the CM population decreased with further differentiation and the markers are less likely to be detected compared to analyses at week 7–10^67,68^. However, differences can also be attributed to the process of assigning markers differently to certain populations as well as the definition of CM and NPC. The clear differences in markers used to identify both CM and NPC in the various publications indicate the challenge of distinguishing cell populations in early development (Supplementary Table 3). Clearly, both a consensus on the main population markers as well as terminology is required in order to ensure comparability of this complex research and provide a common ground for future studies.

Irrespective of the definition of CM and NPC, both differentiating and pluripotent populations were detected. For instance, Wang, et al.^68^ distinguished two transcriptionally distinct populations, which are in line with earlier mouse^18,69–72^ and human studies^73^ investigating the role of *SIX2* and *MUC1* expression in the progenitor pool, respectively. One such population expressed markers *SIX2*, *EYAI,* and *COL1A1*, which are involved in the induction of CM and associated with the self-renewal capacity of stem cells indicated by high *HMGA1* and *HMGA2* expression^68^. Pseudo-time analysis suggested the self-renewing population sustained its proliferative capacity throughout nephrogenesis^68^. The second population was identified as gradually going through MET and expressing epithelial markers like *CLDN11* as well as *NPHS2*, indicating the onset of podocyte differentiation. The fact that Hochane, et al.^59^ detected a self-renewing cluster clustered as NPCs could also indicate a gradual differentiation from CM to NPCs.

In terms of novel markers of the CM, Hochane, et al.^59^ described *UNCX* as a novel marker for the *CITED1*^*+*^ self-renewing population after validating their transcriptomic data with immunostainings. Although UNCX was found in early mouse studies^74–76^, where it was described as a transcription factor involved in distal RV differentiation that disappears around E17.5, this seems to be the first study showing UNCX gene and protein expression in human tissue. Future studies might consider investigating its role by analyzing spatiotemporal expression along with common CM markers in human tissue. In summary, various scRNA sequencing studies in renal development support the presence of a self-renewing and differentiating population within the CM, while distinct NPC populations seem yet to emerge, and the determination of specific cell types is challenging.

### Segmentation and differentiation in early stages of developing nephrons

As development continues, the relative expression of CM genes decreases sharply after approximately week 10 with further cellular differentiation of NPCs and morphological organization into PTAs (Fig. 1). At this stage in mice, PTAs are already well known to be segmented into proximal and distal segments^77^. Three-dimensional imaging of the developing human kidney revealed that NPCs already assign to certain lineages at the onset of the PTA. SIX1^+^ NPCs segment into two layers upon recruiting, where the earliest recruited NPCs are of distal lineage and the latest are of proximal lineage. The last NPCs recruited are hypothesized to be of parietal lineage. The spatiotemporal location of NPCs within the early developing nephron could thus have an impact on their subsequent respective lineage. The latest research from the same group confirmed three distinct populations within the PTA-expressing proximal and distal markers^78^.

Simultaneously with NPC recruitment, the connection between NPC and PTA is gradually reduced until the late renal vesicle stage when it is broken and the SIX2^+^ CM remains on the tip of the UB^66^. In this process, the PTA undergoes MET, polarizes, forms a lumen and cadherin-mediated cell–cell contacts emerge to finally result in the RV. Earlier research shows that the RV is segmented into proximal and distal parts in mice and shows priming for podocytes, parietal epithelial cells, proximal tubules (PTs), and distal tubules (DTs)^79^. Few scRNA sequencing studies of human tissue detected the RV and only one detected more than one cluster, namely five in total, which were defined by patterns of *CDH1*, *JAG1,* and *WT1* expression^78^. Since the latter study sequenced the nephrogenic zone only, it is clearly a challenge to detect the RV or even distinguish populations within whole kidneys.

In the final stage of nephrogenesis, the SSB stage, further segmentation of the nephron starts to be noticeable. Divided into proximal, medial, and distal segments, each segment differentiates further into distinct epithelia^79^. Few scRNA sequencing studies identified the SSB. Like in RVs, *MAFB* expression was associated with the podocyte progenitors within the proximal segment^59^. Correlation of scRNA sequencing and immunofluorescence confirmed the presence of precursors of distal, proximal, LoH populations and renal corpuscle within the SSB^66^. In a preprint, Lindström, et al.^78^ identified six distinct populations by three-dimensional spatial mapping of single-cell transcriptomes, which agreed with the previously identified populations and additionally detected parietal epithelium, CNT, and macula densa/LoH. Interestingly, a single tubular progenitor population initially exists next to podocytes and parietal epithelium. As development proceeds, this tubular population differentiates further into distal and medial domains. Congruence in gene expression with the distinct adult cell types supports the assumption that these early populations are precursors that start to express some transcription factors and genes associated with specific cell type functionality as known from mature cells.

To conclude, while the SSB is distinguished by some scRNA sequencing papers, it clearly remains challenging to distinguish PTAs, RVs, and CSBs. Identification by morphology and correlation to their respective transcriptome was done to overcome this challenge. This is an ideal example of the difficulty of distinguishing cells with subtle differences in the transcriptome following a gradual differentiation during development. However, the preprint of Lindström, et al.^78^ indicates that better molecular and temporal resolution could be achieved by spatially mapping transcriptomes. Alternatively, pseudo-depth analysis could be applied. Furthermore, in combination with markers for the G2/M phase of mitosis, a distinction might be made between RV and CSB based on an assumption of reduced proliferation after the RV stage^59^. By fine-tuning the in vitro replication of kidney development based on such data, more diverse cell types could be generated according to natural lineage trajectories.

### Mesangial cells in development and adulthood

The stromal population comprises a diverse cellular composition. Functionally, it guides nephrogenesis and provides essential signaling around mature nephrons. However, the diversity and functionality of stromal cells are not fully unraveled. ICs, MCs, juxtaglomerular cells, fibroblasts, pericytes, and smooth muscle cells belong to the stromal populations, and all derive from a common FOXD1^+^ precursor^80^. MCs, nowadays considered a special type of pericyte, are located within the glomerulus in direct contact with glomerular endothelial cells, while extra-glomerular endothelial cells are located at the stalk^81,82^. Less than half of the discussed scRNA sequencing publications detected a mesangial cluster, of which just one discriminated intra- and extraglomerular mesangium^68^. This is because the molecular signature of e-MCs is not yet well defined and distinguishing them from intraglomerular MCs (i-MCs) is only possible by analyzing the microanatomy. This correlation of the two techniques has led to interesting results.

Single-cell RNA sequencing revealed that e-MCs had higher proliferative potential, whereas i-MCs were more mature. Interestingly, until week 10, the expression of the mesenchymal cell marker *PDGFRb* and endothelial cell marker *CD31* were restricted to the stromal compartment and later migrated into the glomeruli^68^. This migratory phenomenon has been described in rat development^83^ and aligns with the investigations of Menon, et al.^61^ who presented the expression of *TAGL* in migratory MCs. The molecular distinction of i-MCs and e-MCs was resolved via the co-expression of *PDGFRb* and *LAM4A* of i-MCs. Furthermore, *PIEZO2*, a gene encoding a stretch-gated ion channel, was identified in i-MCs of adult human kidneys. Stretch-gated ion channels in MCs were already described in 1989^84^, but the research is very limited and *PIEZO2* has not been described earlier in this context.

While it is beyond the scope of this review to discuss all stromal cell types in detail and few papers resolved a variety of stromal populations, we would like to highlight recent scRNA sequencing publications investigating the interstitial heterogeneity in both rodent^85^ and human kidneys^86,87^. A brief comparison with human datasets confirmed the conservation of this heterogeneity. Given the important role these cells play in extracellular matrix deposition and endocrine signaling, we hope that future research will continue in-depth characterization.

### Development and maturation of glomerular cells

Pseudo-time trajectories suggest that podocytes are the first differentiated cell type to emerge and mature along a complex genetic trajectory^61^. Remarkably, 228 genes are involved only for podocytes to develop from SSB podocytes to mature podocytes^59,61^. Comparing different gestational weeks showed that podocytes emerge around week 9 of human development^88^. The podocyte-specific markers *MAFB* and *TCF21* are first detected in renal vesicles overlapping with *SIX2* and *TMEM100*^65^. Accordingly, nephron progenitor markers *SIX2*, *EYA1,* and *MEOX1* are downregulated over time and diminish with the onset of early podocyte markers *MAFB* and *TCF21*^65,66^. The developmental trajectory becomes increasingly complex as it continues from this point.

Four studies independently provided evidence for a transitional, immature podocyte population^59,61,65,89^. A small subpopulation of cells, located in the visceral part of the proximal segment of the SSB, follows the podocyte trajectory while expressing a distinct set of markers in the SSB phase until the capillary loop phase^59^. While not all studies agree on all markers of either immature or mature podocytes (Table 2), there is a general consensus that the transitional, immature podocytes express *OLFM3*, and do not express well-known mature podocyte markers such as *NPHS1*, *NPHS2,* and *PTPRO*. Within each of these independent studies, the evidence confirms these findings as additional techniques such as single-molecule FISH and immunofluorescent labeling delivered supporting information^59,65,66^. The marker heterogeneity between the studies of this transient population could be due to age differences of the individuals, technical differences, and the spatial heterogeneity caused by the gradually increasing maturity towards the medulla. However, all except one study detected *OLFM3* expression, confirming that the different studies found the same transitional podocyte cluster.

**Table 2:** Overview of the number of clusters of podocyte progenitors and mature podocytes with their respective markers discovered using single-cell RNA sequencing of developing human kidney.

Author	Tissue age	Podocyte progenitor clusters	Markers early podocytes/ transitional cells	Podocyte clusters	Markers mature podocytes
Hochane, et al. ^59^	w9, w11, w13, w16, w18	1	*OLFM3*^+^/*MAFB*^+^/*FOXC2*^+^/*CLIC5*-^LOW^/ *PTPRO*-^LOW^, *NPHS1*-^LOW^, *NPHS2*-^LOW^	1	*MAFB*-^LOW^, *NPHS2*^+^, *PTPRO*^+^, *PODXL*^+^
Lindstrom, et al. ^66^	w17	1	*CLDN5*^+^, *OLMF3*^+^	1	*TCF21*^+^, *NPHS2*^+^
Lindstrom, et al. ^64^	w16	1	*PODXL*^+^, *MAFB*^+^, *NPHS2*^+^	0	N/A
Menon, et al. ^61^	w12.4, w15, w15.7, w16.4, w18.8	1	*OLFM3*^+^/*MAFB*-^LOW^	1	*PODXL*^+^, *NPHS1*^+^, *NPHS2*^+^, *CLIC5*^+^
Combes, et al. ^89^	w16	1	ON1 CTGF+, *OLFM3*^+^, *MAFB*^+^, *NPHS1*^+^, *LHX1*-^LOW^, *PAX8*-^LOW^	2	*PTPRO*^+^, *SYNPO*^+^, *VEGFA*^+^, *WT1*^+^
Tran, et al. ^65^	w15, w17	1	*LHX1*+, *PAX8*^+^, *FBLN2*^+^, *OLFM3*^+^, *PCDH9*^+^, *SLC16A1*^+^, *GFRA3*^+^, *LEFTY1*^+^	2	*PLA2R1*^+^, *ARMH4*^+^, *F3*^+^, *SYNPO*^+^, *NPHS2*^+^, *MAFB*^+^, *TGFBR3*^+^, *COL4A3*^+^, *COL4A4*^+^, *TNNT2*^+^, *PLCE1*^+^, *ANXA1*^+^
Wang, et al. ^68^	w7, w8, w9, w10, w13, w19, w22, w24, w25	0	N/A	1	N/A
Stewart, et al. ^88^	w7-16	0	N/A	1	*NPHS2*^+^, *PTPRO*^+^, *WT1*^+^, *TCF21*^+^, *PODXL*^+^
Young, et al. ^67^	w8,9	0	N/A	0	N/A
Lindström, et al. ^78^	w14	1	*EFNB2*^+^, *BMP4*^+^, *OLFM3*^+^, *MAFB*^+^	1	N/A

*w* weeks, *N/A* information not available. Gene symbols in italic.

Within the immature podocyte cluster, *OLFM3* was most highly expressed compared to other genes, but diminished towards the CLS with the onset of mature podocyte markers. Proliferation markers had a low expression, which is expected for podocytes^59,90^. To date, no other publication has shown either *OLFM3* expression or a transitional podocyte population in human kidneys. The only transitional cell type related to podocytes was described as CD133^+^/CD24^+^ progenitors, which are located at the urinary pole within the Bowman’s capsule and the PT and have been attributed to possess progenitor-like behavior by transdifferentiating into podocytes in case of injury^91–93^. However, this progenitor-like cell type is not to be confused with the *OLFM3*^+^ population as the latter is a transient population in development, while the former was found to be resident in adults.

Interestingly Brunskill, et al.^94^ extensively mapped the molecular signature of podocytes (144 podocyte-specific genes) in developing and adult mice and found a distinct set of podocyte-specific genes enriched in immature podocytes, with more mature markers emerging over time. These drastic gene expression changes can be associated with both the considerable morphological^95^ and functional changes in podocyte development, where the immature *OLFM3*^+^ population could represent pre-functional podocytes. Combes, et al.^89^ and Tran, et al.^65^ distinguished an additional maturing podocyte cluster that follows the transitional *OLFM3*^+^ subcluster. This might indicate even more complex developmental stages. Markers related to microtubule modulation, such as *TUBA1A* and *STMN1*, were upregulated in this additional cluster, indicating the extensive morphological transformations known for podocyte development^96^. The field would benefit from more in-depth studies elucidating the role of OLFM3 in the maturation of podocytes to support replication of differentiation in vitro.

Another interesting glomerular cell type is the PEC. Terminally differentiated PECs are located as a monolayer on top of the Bowman’s capsule and retain the capacity to proliferate. A variety of distinct PECs have been described according to the expression of different proteins, such as Pax-2 and claudin-1, but also their localization on the Bowman’s capsule^97^. Additionally, PECs have frequently been a discussion point in terms of their transdifferentiation into podocytes in glomerular diseases^91,92,97–99^. Single-cell RNA sequencing research on the nephrogenic zone showed the existence of a common progenitor of PECs and podocytes until the RV stage^78^. A few weeks later in development, sequencing of the whole kidney showed distinct gene expression profiles for PECs and podocytes in a different study^61^, indicating distinct maturation pathways. The potential to transdifferentiate has not been shown in the scRNA sequencing papers. We anticipate that the common progenitor is a starting point for more sophisticated research to investigate the role of PECs in injury and disease.

Surprisingly, only one of the published (single-cell) RNA sequencing papers could detect more than one PEC population; however, characterization of these clusters is still needed^86^. A few other papers found a single cluster of PECs in either fetal kidney^61,65^ or adult kidney^100,101^ with enhanced expression of *CAV2*, *PTRF* (*CAVIN1*), *CLDN1*, *CLDN3*, *LIX1*, *CDH6* and *KRT8*, *KRT18*, *CD24*, *VCAM1* respectively. However, there was no discrimination between subpopulations, while at least two transitional cell types (in between PECs and podocytes) have been described in earlier research^97^. Therefore, future research would benefit from defining the molecular signature of PECs to unravel their complete lineage trajectory and functionalities in adulthood.

### Patterning of tubular epithelial cells

From around week 12 onwards, more cell types are detectable, such as PT, LoH, CD, and PE^17,88^. As previously described, PT and DT differentiate around the same stage and subsequently LoH and CNT emerge from the DT. While little information is provided on tubular maturation within the scRNA sequencing papers, interesting findings provide insight into segment transitions and molecular signatures related to cell functions.

Generally, little is known about the molecular signature of the distinct PT segments. The proximal convoluted tubule (PCT) includes the segments S1 (early PCT) and S2 (late PCT). The proximal straight tubule (PST) is composed of the S2 (cortical PST) and S3 (medullary PST). All scRNA sequencing studies of adult kidney detected at least one PT cluster in adult tissue, of which about half found distinct populations that correlated with the different segments S1–3. However, earlier research has indicated a lack of discrete transitions in morphology in between the distinct segments and the likelihood of intermediate cell types^102^. A continuum of gene expression, as shown in similarity weighted nonnegative embedding (SWNE) analysis, confirmed the existence of such continuous transitions from PT to DCT^103^. A recent publication distinguished seven PT populations of which three expressed markers of both S1 and S2, also confirming a gradual transition^86^. This phenomenon of gradual transition could also explain why some papers resolved only a single PT population^101^.

Single-cell RNA sequencing provides more insight into both molecular anatomy and (patho)physiology. With the PT being the most abundant cell type in the kidney, 54 transcription factors were discriminated, many of which are restricted to the PT^104^. Although markers and transcription factors are identified in various studies^100,104^, it is believed that differential gene expression along the PT trajectory indicates variation in metabolic processes and transport in the various segments^103^. Interestingly, next to metabolic markers, deep sampling revealed marker expressions with a role in immune defense against pathogens, as well as inflammation and regeneration following kidney injury^103^.

The DCT can be distinguished from the PT by a decrease in fatty acid, glucose, amino acid, and hormone metabolism^68^. Furthermore, the distal tubule, LoH, and CNT are thought to derive from the same distal precursor within the SSB, which is distinct from the PT precursor^78^. Only one scRNA sequencing publication distinguished more than one DCT population^101^, although previously two distinct populations have been confirmed. This might indicate a gradual transition between DCT cell types^103^. In contrast, there are indications for an abrupt transition of the thick ascending LoH into the DCT since there was no overlap of the segment-specific markers SLC12A1 and SLC12A3^101^. Indeed the same lack of double-positive cells could be seen in the data set of, for example, Lake, et al.^103^. Morphologically an abrupt transition was already shown in rodents several decades ago^105^.

The CD consists of the cortical collecting duct (CCD) and outer medullary collecting duct (OMCD) and comprises intercalated (ICs) and principal cells (PCs)^102^. Principal cells express the epithelial sodium channel (ENaC). Currently, the classification of intercalated cells takes into account the expression of the chloride–bicarbonate exchanger SLC4A1, the multi-subunit H^+^-ATPase, and pendrin (subtype of the chloride–bicarbonate exchanger). Consequently, IC-A cells apically express H^+^-ATPase, basolaterally express SLC4A1, and lack pendrin. IC-B express H^+^-ATPase at their basolateral pole and non-A, non-B cells express both H^+^-ATPase and pendrin at their apical membrane.

Non-A, non-B cells are, amongst others, located in the CNT^106^, which, against earlier belief, develops from the distal tubule and not the CD in both humans^78^ and mice^79^. The cellular complexity of the CNT could, however, only partly be resolved in the discussed papers. Only one CNT cluster has been reported throughout the studies, although the CNT is known to contain cell types with DCT2 and CD phenotypes^103^. This leads to the question of the identity of this single CNT cluster and why further clustering was or could not be shown. Future studies could include spatial information in their quest to distinguish subpopulations of the CNT and use spatial transcriptomics such as the Nanostring Whole Transcriptome Atlas (WTA) technology.

Nearly all scRNA sequencing papers of adult human kidney detected intercalated cells and most distinguished multiple populations and principal cells. Particularly, single nucleus RNA sequencing disclosed a remarkable resolution of the transitions from DCT to CD by distinguishing two PC and three IC populations. Within these transitions, IC-B, IC-A1, and PC-1 dominated within the cortex, while PC-3 and IC-A2 were highly abundant in the medulla. All IC and PC clusters expressed markers known for the collecting duct^103^. It remains to be determined if PCs and ICs located in the CNT can be distinguished on a transcriptional level. In the recent publication by Kuppe, et al.^86^ a surprising eight distinct IC populations were detected in adult kidneys. Future research could investigate if these clusters contain ICs located within the CNT. In sum, various scRNA sequencing papers succeeded in detecting more than the known cellular variety of populations within the CD, but limited populations within the CNT, which will be highly interesting to characterize further.

Interestingly, the PC marker *AQP2* was also expressed in a subpopulation of IC-A, here named IC-A2, in two independent studies of human kidney^68,103^ as well as in mice^107,108^, leading to the hypothesis that IC cells derive from the first emerging PC cell population with a double-positive transitional/intermediate stage (Fig. 2). More characterization of the IC-A2 cell type is needed, particularly in terms of localization of SLC4A1 and H^+^-ATPase, to clearly identify this cluster since earlier research did not distinguish different IC-A populations^106^. No non-A, non-B cells were detected, which were previously defined as expressing both H^+^-ATPase and pendrin at their apical membrane and to be located in the CNT^106^. The detection of the additional IC subtype IC-A2 could inspire future research to investigate the lineage relationship between ICs and PCs.

All cell populations found in the discussed scRNA sequencing publications are summarized in Supplementary Table 1. Comparing the number of clusters for each cell type as described in each publication (excluding unassigned clusters such as “proliferating”, “differentiating” and too general clusters such as “epithelial cells”) provides insight into the cellular composition of developing and adult kidneys. Overall, 6–19 distinct cell populations of renal lineage were detected across the publications in the developing kidney and 6–33 distinct populations in the adult kidney, with a median of 14 populations in development and 13 in adult kidneys. Between 2–9 distinct non-renal populations were described, with a median of 4 in development and a range of 4–23 with a median of 7 in adult kidneys. Multiple factors could lead to these ranges including different tissue sources (biopsy, resection, whole kidney, etc.), tissue location (cortex vs medulla) or different digestion methods. Therefore, having the papers complement each other’s findings by taking the sum of all different populations of all publications, likely provides a more realistic number. Thus, we counted the largest number of clusters for specific cell types (i.e., seven PT clusters described by Kuppe, et al.^86^) and with this, the total sum was calculated. This approach helps us to provide an approximation for the variety of cell types within human kidneys of both renal and non-renal lineage; namely in total of 73 distinct populations in adult kidneys. Of this, 41 are populations of renal lineage and 32 are populations of non-renal lineage. (Fig. 3) The fetal kidney comprised approximately 32 cell populations of renal lineage; however, developing cells were likely counted repeatedly throughout the various stages of nephrogenesis. Clearly, these numbers are only an approximation of the true variety of cell types, due to the large variation in factors like tissue source, tissue age, dissociation method, terminology, and computational settings. Certain cell types, such as the large variety of stromal cells, are underrepresented in the current research and would need to be added. Furthermore, the definition of what defines a distinct cell type is yet unclear and heavily affects the number of identified cells. However, future studies with further developed protocols and novel technologies will certainly identify additional cell types, which cannot yet be resolved.

## General discussion

In this review, we discuss new knowledge on the cellular composition of healthy developing and adult human kidneys based on scRNA sequencing studies. ScRNA sequencing has the unique potential to provide comprehensive information on the cellular and molecular complexity of various tissues. Therefore, we aimed to compare the current literature in the field to estimate the heterogeneity of kidney cell types. We conclude that scRNA sequencing is a valuable tool to detect a variety of cell types; for instance, transient cell populations such as developing podocytes and a new intercalated cell population.

However, there are also various issues with this technology such as the sparsity of the generated data. For instance, the biological sex of the studied patients was not considered in the analyses and has not been studied elsewhere on a single-cell transcriptomic level. Adding this could be of valuable impact. Differences in function, morphology, and responses to injury are already known to exist between females and males in human developing and adult kidneys^23,109,110^. Recent scRNA sequencing of rodent kidneys also reveals transcriptomic differences between sexes^111^. For instance, markers of PT functionality, including organic anion, amino acid transport, and drug and hormone metabolism, were differentially expressed in female and male mice. Future research could investigate conformity in humans and particularly elaborate on differences in the developmental trajectory in pseudo-time. Additionally, the variety of cell types detected with scRNA sequencing is strongly determined by factors such as computational settings, dissociation methods, tissue source, markers used to identify clusters, and patient age. Standardized reporting of factors such as gestational age would facilitate the integration of multiple datasets and allow further analysis of, for example, transcriptome development in relation to gestational age.

Certain differences of used terminologies, technical limitations, and the lack of the definition of a cell type impede the data comparability and integration. Several comprehensive reviews have been published on solving technical limitations of scRNA sequencing, such as distinguishing rare transcripts from noise, dropouts and computational challenges such as user-defined clustering^42,112,113^.

While many limitations are being addressed, additional techniques will be needed to confirm cellular identity besides the transcriptomic profile. These techniques could be applied to answer research questions in terms of the previously discussed three pillars of cellular identity. They could include anatomical localization of the scRNA sequencing markers by in situ hybridization, in situ sequencing, and studying ligand–cell interactions^114^. Determining cellular functionality and state in response to stimuli in human tissue will primarily require explantation since cell types outside their native environment, in 2D culture, will probably lose their phenotypic characteristics. Clearly, there is still no straightforward definition of a cell type, however, a combination of the aforementioned techniques and models could make a large step towards an atlas of functional human kidney cell types.

Discrimination of early nephrogenic stages and cell type precursors using scRNA sequencing appeared to be consistently challenging throughout various studies due to the subtle molecular and spatial changes. Comparing different studies is particularly difficult, since the transcriptome significantly changes during differentiation, and thus the time point of cell isolation is very important. Consequently, additional validation experiments are needed e.g., using staining of identified markers. Alternatively, differentiation paths in development could be regarded more as a continuum rather than distinct clusters^115^. For instance, podocytes would consequently not be distinguished as separate RV-, CSB-, SSB-podocytes, but as podocytes expressing gradients of transcriptomes throughout development. For this, trajectory interference could be a more suitable method. As such, pseudo-time trajectory analysis has shown that podocytes differentiate prior to the differentiation of tubular cell types and that there is a distinct differentiation pathway for proximal and distal tubular segments. Finding master regulators along these differentiation pathways can support both the understanding of disease as well as in vitro replication of development. Additionally, more insight could be generated into much earlier development on how the degeneration of temporary pro-and mesonephros could influence the pattering of the permanent metanephric kidney.

Sequencing cells from distinct anatomical locations, such as the nephrogenic zone as shown in various publications of Lindstrom, et al.^66^, can help to achieve a higher resolution of the early nephrogenesis. This approach might then also lead to the detection of the cells of the CLS. To conclude, the scRNA sequencing studies on human kidneys to date have shown a detailed transcriptomic complexity and future research will likely discover more.

### Future clinical relevance and impact on regenerative medicine

ScRNA sequencing studies have already given us unprecedented insights into renal development and disease and the technology holds the promise to answer several longstanding questions in the field of nephrology. The higher resolution of molecular mechanisms could provide more insight into complex renal diseases^86^. Cell type-specific gene expression related to chronic kidney disease, diabetes and hypertension could for instance help to identify cell-specific and disease-specific targets and guide the development of urgently needed targeted therapeutics^103^. The diagnosis and treatment of various kidney diseases could be improved based on molecular data of research on pathogenic mechanisms and potential biomarker discovery^43^. ScRNA sequencing is already getting more patient-oriented by the possibility to sequence a variety of kidney cells from urine^116^.

Basic research to develop kidney grafts, such as kidney organoids from induced pluripotent stem cells for future transplantation rely on data on molecular signaling pathways of developing tissue. Currently, these models often lack certain cell types and segments such as the collecting duct, MCs and PECs. More information on the molecular pathways of these cell types is needed in order to reproduce these in vitro. ScRNA sequencing research on developing kidney can provide such data. Additionally, the transcriptome of developing kidneys and in vitro models can be compared as previously shown^104^ to confirm the formation of the appropriate cell types, cellular maturity, and timing. A more comprehensive discussion on this topic can be found in the recent review of Wu and Humphreys^117^.

## Conclusion

In conclusion, we extensively discussed findings of all identified scRNA sequencing publications to date of healthy human developing and adult kidneys and compared their outcome in terms of differences in cellular variety. Together, the publications detected approximately 41 distinct cell populations of renal lineage in the adult human kidney. However, to determine a definite number of cell types, a clear definition of a cell type should be determined and various limitations of the technique need to be resolved. Due to the subtle and gradual changes in the transcriptome in development, we suggest that cells in developing kidneys may best be regarded as a continuum instead of distinct cell types. Detecting master regulators will then help to guide in vitro work along the same differentiation pathways. In the future, advanced technologies such as combined scRNA sequencing and measurement of DNA accessibility, increasing depth, as well as improved analysis methods will likely improve our understanding of the variety of cell types in the adult and developing nephron. Nevertheless, the studies to date provide essential insight into the molecular signature of a large variety of cell populations in adulthood and in development and therefore enabled us to present an updated lineage tree for cell types of renal lineage. On a final note, we trust that these studies will shape the future of regenerative medicine by unraveling lineage trees at an even higher resolution and thus help in organoid and tissue engineering approaches.

## Supplementary information

Supplementary Information
