# Supplementary material for: Defining the variety of cell types in developing and adult human kidneys by single-cell RNA sequencing
Source: NPJ Regen Med. 2021 Aug 11;6:45. doi: 10.1038/s41536-021-00156-w (PMC8357940; doi:10.1038/s41536-021-00156-w)
Supplement: Supplementary file 1 — Supplementary Information [file 41536_2021_156_MOESM1_ESM.pdf]

Supplementary Table 1

Author	Date	Age	Sum of all MM and UB derived clusters/cell types	Sum of all non-renal clusters/cell types
Hochane	2019	Fetal	19	3
Wang	2019	Fetal	10	4
Lindstrom 1	2018	Fetal	15	7
Young	2018	Fetal	6	2
Lindstrom 2	2018	Fetal	10	6
Stewart	2019	Fetal	12	9
Menon	2018	Fetal	18	4
Combes	2019	Fetal	13	6
Tran	2019	Fetal	18	3
Lindstrom (preprint)	2020	Fetal	18	0
Count median cluster number				
Count max renal cell types found				**32**
Count max clusters found				47
Difference = max # non-renal cell types				15
Median			14	4
Range			6-19	2-9
Wu1	2018	Adult	13	4
Wu2 - transplant biopsy	2018	Adult	7	9
Wu2 - healthy kidney		Adult	6	0
Young	2018	Adult	15	4
Stewart	2019	Adult	13	13
Sivakamasundari (preprint)	2017	Adult	11	12
Liao	2020	Adult	8	3
Kuppe	2020	Adult	33	23
Lake	2019	Adult	19	7
Range			6-33	4-23
Median			13	7
Count median cluster number				
Count max renal clusters found				**41**
Count maximum clusters found				73
Difference = max # non-renal cell types				32

Early developmental stages / Progenitors

Author	CM	UB	PTA	PTA/RV	RV	CSB/SSB	RV/CSB	SSB	SSBm/d	SSB prox.	SSB dist.	NPC	Proliferating NPC	Prolif-com/prim NPC	Primed NPC
Hochane		1	1				2		1	1		4			
Wang	2														
Lindstrom 1				2								4	1		2
Young	2	1			1										
Lindstrom 2												1	1		2
Stewart	2	1			1	1									
Menon	1		2					1			1	1			
Combes												1			1
Tran		1										1	1	1	1
Lindstrom (preprint)			3				5	6				2		2	
Count median cluster number															
Count max renal cell types found	2	1	3				5	6							
Count max clusters found												Not added to the calculation as these as they are likely same as CM, PTA, RV, CSB, SSB cells			
Difference = max # non-renal cell types															
Median															
Range															
Wu1															
Wu2 - transplant biopsy															
Wu2 - healthy kidney															
Young															
Stewart															
Sivakamasundari (preprint)															
Liao															
Kuppe															
Lake															
Range															
Median															
Count median cluster number															
Count max renal clusters found															
Count maximum clusters found															
Difference = max # non-renal cell types															

Precursors / Stroma

Author	E-PC	PT precursor	PD precursor	DT precursor	INT precursor	INT	Prolif. INT	FIBRO	MYO-FIB	Juxtaglom	PERI	MES	I-MES	E-MES
Hochane			1		1	2						1		
Wang						1							1	1
Lindstrom 1		1	1	1										
Young								1	1					
Lindstrom 2			1			5								
Stewart								1	1					
Menon			1			2						1		
Combes			1			6								
Tran			1			4	1			1		1		
Lindstrom (preprint)														
Count median cluster number														
Count max renal cell types found								1	1	1			1	1
Count max clusters found														
Difference = max # non-renal cell types														
Median														
Range														
Wu1												1		
Wu2 - transplant biopsy								1	1		1			
Wu2 - healthy kidney														
Young								1	1					
Stewart	1							1	1					
Sivakamasundari (preprint)												1		
Liao						1								
Kuppe								3	2					
Lake												1		
Range														
Median														
Count median cluster number														
Count max renal clusters found	1							3	2		1	1		
Count maximum clusters found														
Difference = max # non-renal cell types														

Mature nephron incl. collecting duct

Author	Epithelium	Distal	Proximal	PD	PECs	PT	PCT	PST	PT S1	PT S2	PT S3	injured PT	LoH	A-LoH	D-LoH	DT/LoH	DT	CNT	PC	IC	TE	CD	Pelvis
Hochane				1		1										1		1					
Wang				1		1							1				1					1	
Lindstrom 1				1		1										1							
Young																							
Lindstrom 2																							
Stewart				1		1							1					1					1
Menon				1	1	1										1		1				3	
Combes		1	1	2																			
Tran				2	1	1																1	
Lindstrom (preprint)																							
Count median cluster number																							
Count max renal cell types found				2	1	1							1				1	1				3	1
Count max clusters found																							
Difference = max # non-renal cell types																							
Median																							
Range																							
Wu1				1					1	1	1			2	1		1	1	1	2			
Wu2 - transplant biopsy						1								1	1							1	
Wu2 - healthy kidney				1		1							1				1		1	1			
Young				1					1	1	1		1				1			2			1
Stewart				1		2							1					1	1	2	1		1
Sivakamasundari (preprint)				1	1	2											4			2			
Liao					1	1	1	1									1		1	1			
Kuppe				1	2			1	3	3		1		3		1	1	1	1	8		1	
Lake	1			1					1	1	2			4	1		1	1	2	3			
Range																							
Median																							
Count median cluster number																							
Count max renal clusters found				1	2	7								4	1		1	1	2	8	1		1
Count maximum clusters found																							
Difference = max # non-renal cell types																							

Ureter / Vasculature/endothelium

Author	Ureter	U1	U2	U3	U4	ENDO	injured ENDO	lENDO	vENDO	aENDO	vSMC	VR	AVRE	DVRE	AEA	DVRE/AEA	PCE	ERY	ER-C	GE
Hochane						1														
Wang						1												1	1	
Lindstrom 1																				
Young						1														
Lindstrom 2						1														
Stewart						1														
Menon						1												1		
Combes						1														
Tran						1												1		
Lindstrom (preprint)																				
Count median cluster number																				
Count max renal cell types found						1												1		
Count max clusters found																				
Difference = max # non-renal cell types																				
Median																				
Range																				
Wu1						1														
Wu2 - transplant biopsy						1														
Wu2 - healthy kidney																				
Young		1	1	1	1								2	1						1
Stewart													1	1			1			1
Sivakamasundari (preprint)											1		1	1	1					1
Liao																				
Kuppe	1						1	1	1	1	1	6								1
Lake						1					1		1			1				1
Range																				
Median																				
Count median cluster number																				
Count max renal clusters found		1	1	1	1			1	1	1	1	6			1		1			1
Count maximum clusters found																				
Difference = max # non-renal cell types																				

Immune/blood/neural cells / Undefined populations

Author	IMM	MACRO	MEGA	pDC	MNP	Mono	Bcells	Tcells	NK/T cells	Leukocytes	NEUTRO	NK	MAST	DC	pDC	Schwann cells	Myeloid	Plasma	Blood	Glia	Ganglion	Cycling/proliferating	Prolif. Primed/diff.	Differentiating	Undefined
Hochane										1												1			
Wang	1																								
Lindstrom 1																						4	1	2	
Young																					1				
Lindstrom 2	1																					2		2	
Stewart		1		1		1	1	1				1	1	1											
Menon	1																		1						
Combes	1																		1	1		2			
Tran	1																								
Lindstrom (preprint)																									
Count median cluster number																									
Count max renal cell types found		1		1		1	1	1		1		1	1	1						1	1				
Count max clusters found																									
Difference = max # non-renal cell types																									
Median																									
Range																									
Wu1		1																							2
Wu2 - transplant biopsy						2	1	1					1					2				1			
Wu2 - healthy kidney																									
Young																									
Stewart					1		1	3			1	1	1		1										
Sivakamasundari (preprint)							1	1				1	1				2	1							
Liao						1	1		1																
Kuppe		3				1	1	1				1	1	1		1		1							
Lake		1																							1
Range																									
Median																									
Count median cluster number																									
Count max renal clusters found		3			1	2	1	3			1	1	1		1	1	2	2							
Count maximum clusters found																									
Difference = max # non-renal cell types																									

Supplementary Table 2

	Date	First author	DOI	Fetal/adult	Embryonic week	Part of kidney digested and analysed	Number of individuals/tissues	Number of cells analysed	Methods	Number of clusters
1	2018	Young	https://doi.org/10.1126/science.aat1699	renal tumor and normal fetal, pediatric and adult	w8,9 Adolescent=12 years	Fetal = kidneys whole dissociation. Adolescent/adult = where possible separate cortical, medullary, pelvic and ureteric biopsies. Otherwise interface of cortex & medulla	2 fetuses, 1 adolescent, 1 adult (and more diseased donors)	Adult kidney: 42,809 nonmalignant cells, 37,951 mature. Fetal kidney: 4858	scRNA seq.	Adult: 19. Fetal: 3 nephron clusters, residual = (myo)fibrobl.,endo., gangl. Adolescent: not stated
2	2018	Menon	https://doi.org/10.1242/dev.164038	fetal	87, 105, 110, 115 and 132 days of gestation	Whole kidney	5	6414	scRNA seq.	11 (8 subclusters within NPCs)
3	2018	Lindstrom 2	https://doi.org/10.1681/ASN.2017080890	fetal	13 to 18 (for scRNA seq w16)	Nephrogenic zone	5 for conventional RNA seq., 1 for sc RNA seq.	scRNA seq: 2750 (318 cells of cluster 4 - NPC)	bulk seq and scRNA seq.	Initially 12, of which 1 cluster NPC related: this divided into 4 clusters (of scRNA seq)
4	2018	Wang	https://doi.org/10.1016/j.celrep.2018.08.056	fetal	w7, w8, w9, w10, w13, w19, w22, w24, w25	w7-10: whole kidney, w15-25: sections	11 (with 1 biol. Replicate of w7 and w19	3023 cells of all 11 embryos	scRNA seq.	13
5	2019	Combes	https://doi.org/10.1186/s13073-019-0615-0	fetal	w16	Did not generate new datasets. Used Lindstrom 2018 dataset and re-analyzed and compared with organoids.	nephrogenic zone of 2 fetuses	N/A. renalayzing scRNA seq. data	N/A. renalayzing scRNA seq. data	16
6	2020	Lindstrom (preprint)	https://doi.org/10.1101/2020.04.27.060749	fetal	week 14	Nephrogenic zone	2	24,254 cells (mean of 2644 genes/cell)	scRNAseq.	18
7	2019	Stewart	https://doi.org/10.1126/science.aat5031	fetal + adult	7-16 PCW	Fetal = whole kidney. Adult = healthy tissue from tumour nephectromy and healthy donor tissue (cortex, medulla, pelvis, ureter)	unknown	40,268 (adult), 27,203 (fetal)	high-throughput scRNAseq, bulk RNA seq	Adut = 25, fetal = 21
8	2019	Tran	https://doi.org/10.1016/j.devcel.2019.06.001	fetal	week 15 and 17 LMP	Vibratome sections, dissected into inner and outer cortex	2	week 17: 7,518 of zone 1 and 2 together. Week 15: 6,129 of zone 1 and 2 together	scRNA seq.	21
9	2019	Hochane	https://doi.org/10.1371/journal.pbio.3000152	fetal	w9, w11, w13, w16, w18	Whole kidney	one kidney per gestational week	wk 16 = 6,602/ w9+w11+w13+w18 = 11,359	scRNA seq.	22 (29 original clusters, similar ones were merged)
10	2018	Lindstrom 1	https://doi.org/10.1016/j.devcel.2018.05.010	fetal	w8 to 18 (but two replicates of week 17 for scRNA seq)	Nephrogenic zone	nephrogenic zone of 2 fetuses	7343 cells with 3367 cells of nephrogenic lineage	scRNA seq.	22
11	2020	Liao	https://doi.org/10.1038/s41597-019-0351-8	adult	x	Whole kidney sections (0.5-1g) from nephrectomy (healthy tissues next to tumor)	3	23,366 (of 3 donors together)	scRNA seq.	10
12	2018	Wu 2	https://doi.org/10.1681/ASN.2018020125	adult	x	Allograft biopsy	single healthy kidney 4 biopsies	4259 nuclei, 4487 cells	scRNA seq.	16
13	2018	Wu 1	https://doi.org/10.1016/j.stem.2018.10.010	adult	x	Cortex of nephrectomy	one kidney	4,524 nuclei	snRNA seq.	17

14	2017	Sivakamasundari (preprint)	http://dx.doi.org/10.1101/238063	adult	x	Kidney resection samples	3	22,469 (initially 3,128, then 19,341 from two donors used for further analysis)	scRNAseq	27
15	2020	Kuppe	https://doi.org/10.1038/s41586-020-2941-1	adult	x	Healthy tumor nephrectomy tissue	11 (of which 7 with GFR>60	53,672 cells	scRNAseq, bulk RNA seq, ATAC seq.	50
16	2019	Lake	https://doi.org/10.1038/s41467-019-10861-2	adult	x	Nephrectomy samples, partly containing both cortex and medulla	15	17,659 nuclei	snRNA seq.	30

Supplementary Table 3

CELL TYPE	Hochane<br>fetal	Wang<br>fetal	Lindstrom1<br>fetal	Lindstrom2<br>fetal	Young<br>fetal	Stewart<br>fetal	Menon<br>fetal	Combes<br>fetal	Tran<br>fetal
**Cap mesenchyme**	N/A	SIX2	N/A	N/A	SIX2<br>CITED1<br>PAX2<br>SIX1	SIX2<br>ITGA8<br>MAFB<br>WT1<br>CITED1<br>SALL1<br>PAX2<br>OSR1<br>WNT4<br>BMP7<br>HOXA11	H19<br>ITGA8<br>NCAM1<br>MEIS2<br>MAFB<br>NR2F2<br>EYA1<br>NTRK2	N/A	N/A
**Nephron progenitors**	OSR1<br>SIX1<br>SIX2<br>CITED1<br>EYA1<br>SALL1<br>MEOX1<br>GDNF<br>ETV4<br>COL2A1<br>HES1<br>CRABP2<br>LEF1<br>ITGA8<br>LHX1<br>WNT4<br>CCND1	N/A	CHRNA1<br>SLC15A1<br>DAPL1<br>TMEM100<br>MEOX1<br>CITED1<br>CRABP2<br>PDK4<br>WASF3<br>MALAT1	CITED1<br>SIX1<br>LYPD<br>DAPL1	N/A	N/A	N/A	N/A	TMEM100<br>CITED1<br>MEOX1

**Pretubular aggregate**		N/A	N/A	N/A	N/A	N/A	EYA1<br>JAG1<br>CCND1<br>LHX1<br>EMX2	N/A	N/A
**Committed/primed nephron progenitor**	N/A	N/A	ID3<br>HEY1<br>LYPD1<br>ID1<br>PAX2<br>ID2	ID1<br>MEG3<br>DAPL1	N/A	N/A	N/A	N/A	MAL<br>CLDN10<br>HES1
**Proliferating nephron progenitor**	N/A	N/A	HIST1H2AC<br>IGFBP5<br>MFAP4	CENPF<br>TOP2A	N/A	N/A	N/A	N/A	TOP2A<br>SIX1<br>TMEM100
**Proliferating committed nephron progenitor**	N/A	N/A	N/A	N/A	N/A	N/A	N/A	N/A	PCNA<br>LHX1<br>PAX2
**Self-renewing NPC**	N/A	N/A	N/A	TMEM100<br>CITED1<br>ROBO2<br>MEOX1	N/A	N/A	N/A	N/A	N/A
**Differentiating NPC**	N/A	N/A	N/A	CCND1<br>PAX8<br>LHX1	N/A	N/A	N/A	N/A	N/A

**Renal vesicle & comma-shaped body**	LHX1<br>PAX8<br>JAG1<br>PAX2<br>WNT4<br>SFRP2<br>DLL1	N/A	N/A	N/A	N/A	N/A	N/A	N/A	N/A
**Proximal renal vesicle**	N/A	N/A	N/A	N/A	N/A	MAFB<br>WT1<br>PAX2<br>FOXC2<br>TMEM100<br>LMX1B<br>NPHS1<br>NPHS2<br>JAG1<br>PAX8<br>NOTCH2	N/A	N/A	N/A
**Distal renal vesicle**	N/A	N/A	N/A	N/A	N/A	LHX1<br>DLL1<br>DKK1<br>FGF8	N/A	N/A	N/A
**Renal vesicle**	N/A	N/A	N/A	N/A	PTPRO<br>PODXL<br>WT1	N/A	LHX1<br>EMX2<br>JAG1<br>DKK1<br>IGFBP4<br>CCND1	N/A	N/A

**PTA/RV**	N/A	N/A	GAD2<br>CCND1<br>KRT8<br>PAX8<br>CXCR4<br>LHX1<br>EMX2	N/A	N/A	N/A	N/A	N/A	N/A
**S-shaped body**	N/A	N/A	N/A	N/A	N/A	N/A	JAG1<br>CCND1<br>LHX1<br>ADAMTS1<br>POU3F3<br>EMX2<br>SIM2<br>DKK1<br>EPHA7<br>EPHA4<br>PLEKHA1	N/A	N/A
**S-shaped body, medial and distal segments**	HNF1B<br>POU3F3<br>SIM2<br>SOX9<br>IRX2<br>IRX3	N/A	N/A	N/A	N/A	IRX1<br>IRX2<br>HNF1B<br>POU3F3<br>JAG1<br>CCND1	N/A	N/A	N/A
**S-shaped body, proximal segment**	CDH6<br>HNF1A<br>AMN	N/A	N/A	N/A	N/A	MAFB<br>WT1<br>FOXC2<br>TMEM100<br>LMX1B	N/A	N/A	N/A

						NPHS1<br>NPHS2			
**S-shaped body, proximal segment (podocyte precursor cells)**	FOXC2<br>MAFB<br>OLFM3<br>WT1	N/A	N/A	N/A	N/A	N/A	N/A	N/A	N/A
**Podocyte progenitor (early podocyte)**	N/A	N/A	OLFM3<br>MAFB<br>CTGF<br>CLDN5	N/A	N/A	N/A	N/A	N/A	PAX8<br>PCDH9<br>OLFM3
**Glomerulus**	N/A	N/A	N/A	N/A	PTPRO<br>PODXL<br>WT1	PTH2R<br>CRB2<br>AQP1<br>EDH3	N/A	N/A	N/A
**Podocytes**	PTPRO<br>NPHS2<br>NPHS1<br>PODXL<br>TGFBR3<br>CLIC5<br>CITED2	NPHS1<br>NPHS2	MAFB<br>AIF1<br>MYL9<br>TCF21<br>PTPRO	N/A	N/A	NPHS1<br>NPHS2<br>PODXL<br>WT1<br>PTPRO<br>CD2AP<br>ITGB5<br>SYNPO<br>CLDN5<br>CLIC5<br>MAFB	PODXL<br>NPHS1<br>NPHS2<br>CLIC5<br>MAFB	N/A	NPHS2<br>MAFB<br>PTPRO<br>CLIC5<br>MAFB<br>PODXL
**Juxtaglomerular cells**	N/A	N/A	N/A	N/A	N/A	N/A	N/A	N/A	REN<br>MEF2C<br>MYH11

**Parietal epithelial cells**	N/A	N/A	N/A	N/A	N/A	N/A	N/A	N/A	LIX1<br>CLDN1<br>CDH6
**Loop of Henle**	N/A	UMOD<br>POU3F3	N/A	N/A	N/A	N/A	SLC3A1<br>CLDN10<br>KCNJ1<br>ATP1B1<br>FXYD2<br>EPHA7<br>TBC1D4<br>POU3F3<br>MECOM	N/A	N/A
**(Thick) ascending limb**	N/A	N/A	N/A	N/A	CLDN16	CLDN16<br>UMOD	N/A	N/A	N/A
**Descending limb**	N/A	N/A	N/A	N/A	SLC12A1	SLC12A1	N/A	N/A	N/A
**Distal tubule & loop of Henle**	PAPPA2<br>MAL<br>CLCN5<br>SLC12A3<br>UMOD<br>SLC12A1	N/A	FXYD2<br>SLC12A1<br>MAL<br>ALDH1A1	N/A	N/A	N/A	SLC12A1<br>UMOD<br>CLCNKB<br>MECOM<br>POU3F3<br>DEFB1 (human)	N/A	N/A
**Distal tubule**	N/A	CLCNKB	N/A	N/A	AVPR2<br>SLC8A1<br>KCNJ1	SLC12A3	FYXD2<br>CA2<br>MAL<br>TBC1D4<br>DEFB1 (human)<br>POU3F3<br>MECOM	N/A	N/A
**Distal precursor**	N/A	N/A	LIMCH1<br>MECOM<br>MAL<br>SOX9	N/A	N/A	N/A	N/A	N/A	N/A

**Early proximal tubule**	LRP2<br>ANPEP<br>SLC34A1<br>CLDN1<br>CLDN2<br>SLC13A1<br>CUBN	N/A	N/A	N/A	N/A	N/A	N/A	N/A	N/A
**Proximal precursor**	N/A	N/A	IGFB7<br>JAG1<br>CDH6<br>TPSAN12	N/A	N/A	N/A	N/A	N/A	N/A
**Proximal tubule**	N/A	LRP2<br>CUBN	C19orf77<br>CUBN<br>SLC3A1<br>GLYATL1	N/A	SLC13A3<br>SLC34A1	FAM151A<br>SLC22A8<br>ALDOB<br>GLYAT<br>SLC13A1<br>DPEP1<br>HNF1A<br>HNF4A<br>HNF4G<br>SLC5A2	SLC3A1<br>CUBN<br>PDZK1<br>LRP2<br>CA2<br>FXYD2<br>ATP1B1<br>ADAMTS1	N/A	SLC3A1<br>LGALS2<br>APOA1
**Distinct proximal tubule**	N/A	N/A	N/A	N/A	N/A	VACM1	N/A	N/A	N/A
**Proximal straight tubule**	N/A	N/A	N/A	N/A	SLC7A13<br>SLC16A9<br>SLC22A7	N/A	N/A	N/A	N/A
**Proximal distal tubule**	N/A	N/A	N/A	N/A	SLC17A3<br>SLC22A8	N/A	N/A	N/A	N/A
**Connecting tubule**	ALDH1A1<br>TACSTD2<br>CDH1	N/A	N/A	N/A	N/A	CALB1	ATP6V1B1<br>FXYD2<br>MECOM<br>TBC1D4<br>PLEKHA1	N/A	N/A

**Interstitial progenitor cells**	GDNF<br>FOXD1	N/A	N/A	N/A	N/A	FOXD1<br>PBX1<br>PDGFRA	N/A	N/A	N/A
**Proliferating interstitial cells**	N/A	N/A	N/A	N/A	N/A	N/A	N/A	N/A	TOP2A<br>COL1A2<br>PCNA
**Interstitial cells**	DES<br>COL3A1<br>COL1A1<br>SERPINE2<br>FGF7<br>DCN<br>LEF1	SFRP1<br>MEIS1<br>PDGFRA	N/A	SULTEI1<br>TCF21<br>ALDH1A2<br>VCAM1<br>FABP2<br>ANGPT1<br>LUM<br>SFRP2<br>DCN<br>MEIS1<br>TAGLN<br>POSTN<br>REN MGP<br>GATA3	N/A	N/A	COL1A2<br>COL3A1<br>TNC<br>COL6A3<br>LGALS1<br>MAFB<br>NR2F2<br>IGFBP4	N/A	COL1A2<br>PDGFRA<br>COL1A1<br>SULT1E1<br>OLFM3<br>ANGPT1<br>PBX1<br>MEIS2<br>COL8A1<br>GUCY1A3<br>TGFBI<br>ZEB2
**Fibroblast**	N/A	N/A	N/A	N/A	SFRP2<br>EMILIN1<br>MMP2	LUM<br>TNC<br>COL1A1<br>COL3A1<br>COL1A2<br>COL1A1	N/A	N/A	N/A
**Myofibroblast**	N/A	N/A	N/A	N/A	PDGFRB<br>ACTA2	PDGFRB	N/A	N/A	N/A
**Mesangial cells**	ACTA2<br>TPM2<br>PDGFRB<br>MCAM<br>CSPG4<br>CD248	N/A	N/A	N/A	N/A	N/A	LGALS1	N/A	TMEFF1<br>GATA3<br>COL4A1

**Collecting duct & Ureteric Bud**	KRT18<br>KRT8<br>RET<br>CLDN7<br>AQP2<br>GATA2<br>MMP7<br>CALB1	QP2<br>AQP3	N/A	N/A	N/A	N/A	N/A	N/A	N/A
**Uteric bud**	N/A	N/A	N/A	N/A	RET<br>GATA3<br>ELF3<br>POU3F3<br>TFCP2L1<br>CDH16	HNF1B<br>GATA3<br>RET<br>WNT9B<br>SOX8<br>SOX9<br>GFRA1<br>WNT11<br>ELF3<br>POU3F3<br>TCFP2L1<br>CDH16<br>KRT8<br>KRT19	N/A	N/A	ALDH1A1<br>RET<br>CRABP1
**Collecting duct**	N/A	N/A	N/A	N/A	ATP6V0D2<br>CLCNKB<br>CLDN8	PVALB<br>CLNK<br>TMEM213<br>CLCNKA<br>CLCNKB<br>KRT8<br>KRT19	KCNJ1<br>GATA3<br>ELF5<br>STC1<br>TBX3<br>CLDN4<br>ATP6V1B1<br>MAL<br>POU3F3<br>TBC1D4<br>KNG1 (rat)<br>CA2<br>DEFB1 (human)<br>PLEKHA1	N/A	KRT18<br>AQP2<br>CALB1
**Intermedullary collecting duct**	N/A	N/A	N/A	N/A	N/A	SLC14A2	N/A	N/A	N/A

**Intercalated cells**	N/A	N/A	N/A	N/A	N/A	FOXI1	TMEM213 (human)	N/A	N/A
**Intercalated cell type A**	N/A	N/A	N/A	N/A	SLC4A1	N/A	SLC4A1	N/A	N/A
**Intercalated cell type B**	N/A	N/A	N/A	N/A	SLC26A4	N/A	SLC26A4	N/A	N/A
**Principal cells**	N/A	N/A	N/A	N/A	AQP2	AQP2<br>AQP3	N/A	N/A	N/A
**Macula densa**	N/A	N/A	N/A	N/A	N/A	PTGS2	N/A	N/A	N/A
**Pelvic epithelium**	N/A	N/A	N/A	N/A	KRT23<br>SAA2	SAA2	N/A	N/A	N/A
**Transitional epithelium of ureter (urothelium)**	N/A	N/A	N/A	N/A	N/A	UPK1A<br>UPK1B	N/A	N/A	N/A
**Pelvis & ureter**	N/A	N/A	N/A	N/A	TP63<br>PVRL4<br>KRT5<br>UPK1B<br>UPK1A<br>DHRS2<br>S100P	UPK3A<br>S100P<br>DHRS2	N/A	N/A	N/A
**Endothelial cells**	KDR<br>TEK<br>FLT1<br>CDH5<br>PECAM1	N/A	N/A	PLVAP<br>GNG11<br>TIE1	N/A	PECAM1	ADGRL4<br>ADGRF5<br>NOSTRIN<br>PECAM1<br>CD34	N/A	PLVAP<br>VAMP5<br>EMCN
**Fenestrated endothelium**	N/A	N/A	N/A	N/A	N/A	PLVAP	N/A	N/A	N/A
**Non-fenestrated vasa recta**	N/A	N/A	N/A	N/A	N/A	SLC14A1	N/A	N/A	N/A
**Glomerular vasculature**	N/A	N/A	N/A	N/A	CLDN5<br>SEMA3G<br>AQP1	N/A	N/A	N/A	N/A

**Vascular**	N/A	N/A	N/A	N/A	PLVAP<br>SLC14A1<br>VCAM1<br>KDR<br>PTPRB<br>PECAM1	N/A	N/A	N/A	N/A
**Leukocytes**	CD37<br>CD48<br>ITGB2<br>IFI30<br>IL1B	N/A	N/A	N/A	N/A	N/A	N/A	N/A	N/A
**Immune cells**	N/A	N/A	N/A	HLA-DRA<br>CCLN3<br>SRGN	N/A	N/A	N/A	N/A	LYZ<br>PTPRC<br>IL1B
**Erythrocytes**	N/A	N/A	N/A	N/A	N/A	N/A	MTCO1P12<br>MTND1P23<br>RPL10P9<br>HBG2<br>HBA2<br>HEMGN<br>AHSP<br>ALAS2<br>HBG1<br>HBB	N/A	HBM<br>HBB<br>HBA1
**Hematopoietic cells**	N/A	N/A	N/A	N/A	N/A	N/A	LST1<br>HLA-DPA1<br>SRGN<br>MINDA<br>CORO1A	N/A	N/A
**Blood cells**	N/A	N/A	N/A	N/A	N/A	N/A	N/A	N/A	N/A
**Proliferating cell**	N/A	N/A	HIST1H4C<br>NUSAP1<br>KIAA0101<br>UBE2C<br>CENPF	HIST1H4C<br>HIST1H1A	N/A	N/A	HIST1H1A<br>HIST1H1B<br>HIST1H1C<br>HIST1H1D<br>HIST1H1E	N/A	N/A

			TOP2A<br>TYMS<br>DUT<br>PCNA						
**Differentiating cell**	N/A	N/A	LYPD1<br>IFIM3<br>HEY1<br>PAX8	MAL<br>ALDH1A1<br>KRT18<br>GATA3<br>CDH6<br>EMX2<br>LRP2	N/A	N/A	N/A	N/A	N/A
**Primed/differentiating cell**	N/A	N/A	CTGF<br>HEY1<br>ID1-3<br>GADD45B	N/A	N/A	N/A	N/A	N/A	N/A
**Prolif. Prim. Differentiating cell**	N/A	N/A	LGALS1<br>MT2A<br>MT1X<br>BIRC5	N/A	N/A	N/A	N/A	N/A	N/A
**Glia cells**	N/A	N/A	N/A	N/A	N/A	N/A	N/A	N/A	N/A
